# Supplementary material for: Highly Orientated Sericite Nanosheets in Epoxy Coating for Excellent Corrosion Protection of AZ31B Mg Alloy
Source: Nanomaterials (Basel). 2023 Aug 11;13(16):2310. doi: 10.3390/nano13162310 (PMC10457806; doi:10.3390/nano13162310)
Supplement: Supplementary file 1 [file nanomaterials-13-02310-s001.zip › nanomaterials-2462468-supplementary.pdf]

Supplementary Information

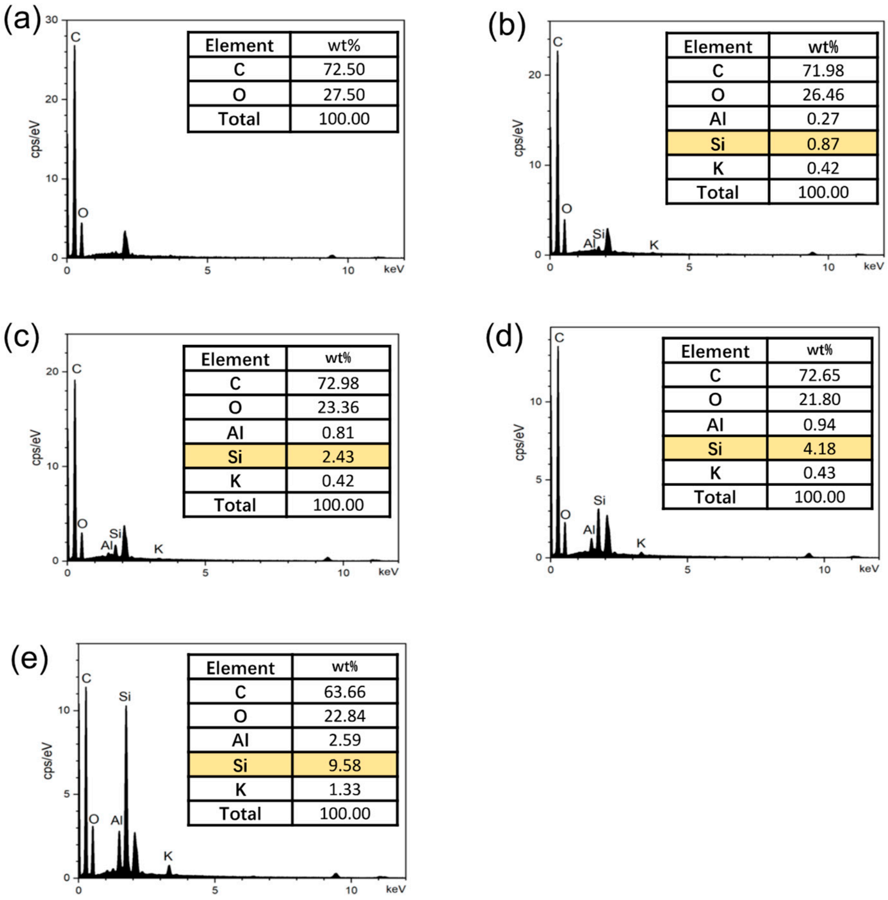

Figure S1. EDS spectra and related elemental concentration obtained from the surface of different samples: (a) E, (b) ES-1, (c) ES-2, (d) ES-4, and (e) ES-6.
